# Supplementary material for: Forward Inverse Relaxation Model Incorporating Movement Duration Optimization
Source: Brain Sci. 2021 Jan 23;11(2):149. doi: 10.3390/brainsci11020149 (PMC7912108; doi:10.3390/brainsci11020149)
Supplement: Supplementary file 1 [file brainsci-11-00149-s001.pdf]

Supplementary Materials: Forward Inverse Relaxation Model Incorporating Movement Duration Optimization

Misaki Takeda, Isao Nambu and Yasuhiro Wada

Supplementary Figures

The results for subjects other than the representative subject A (i.e., subjects B, C, D, E, and F) are shown in Supplementary Figures S1–S5.

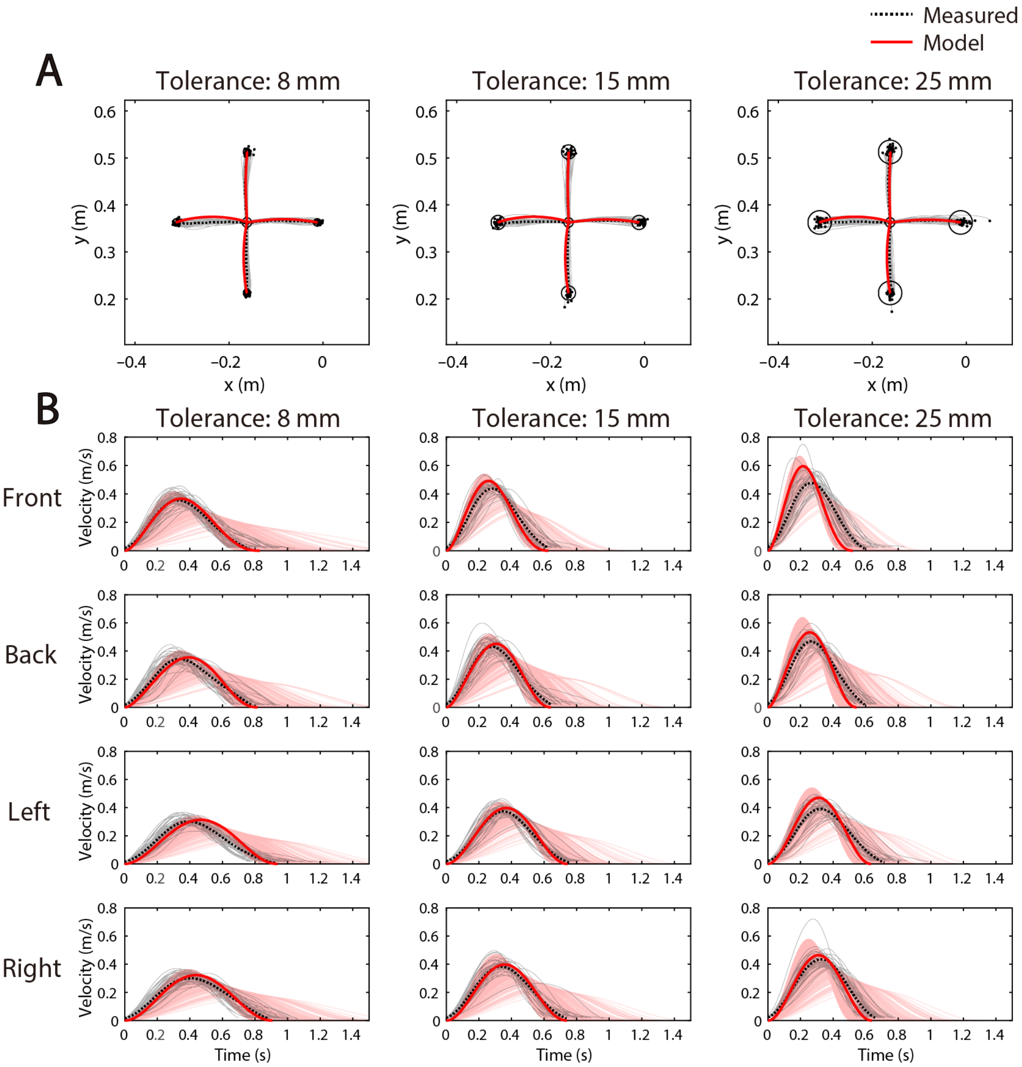

Figure S1. Subject B (Movement duration)

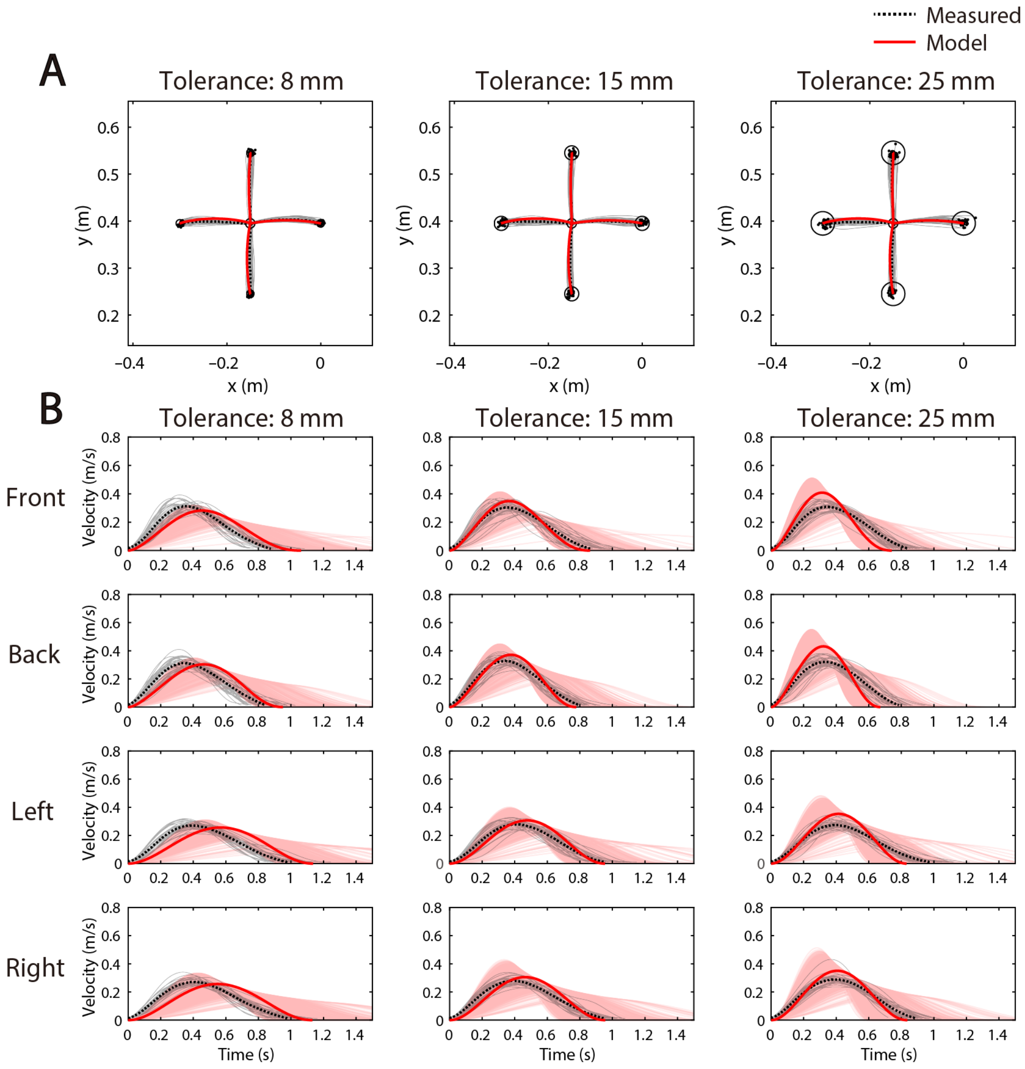

Figure S2. Subject C (Movement duration)

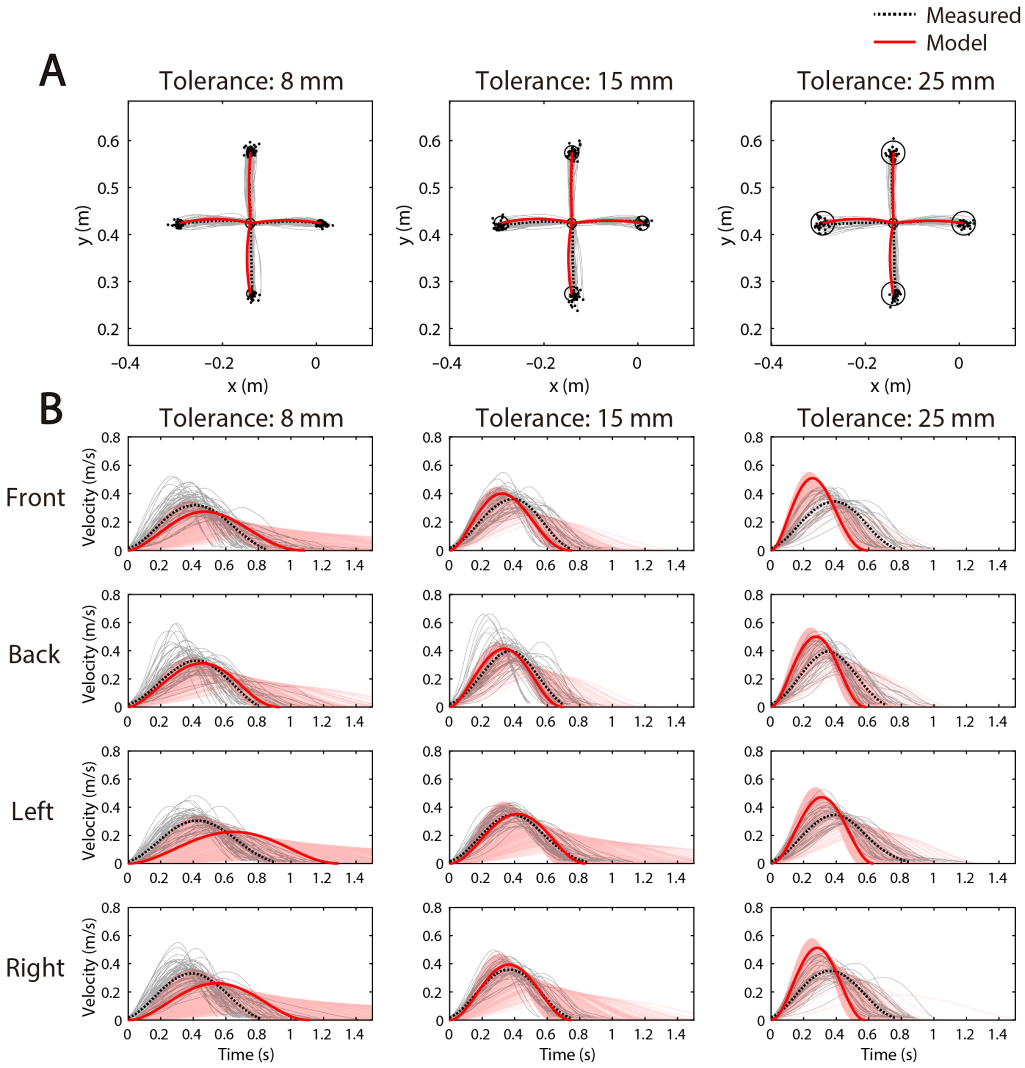

Figure S3. Subject D (Movement duration)

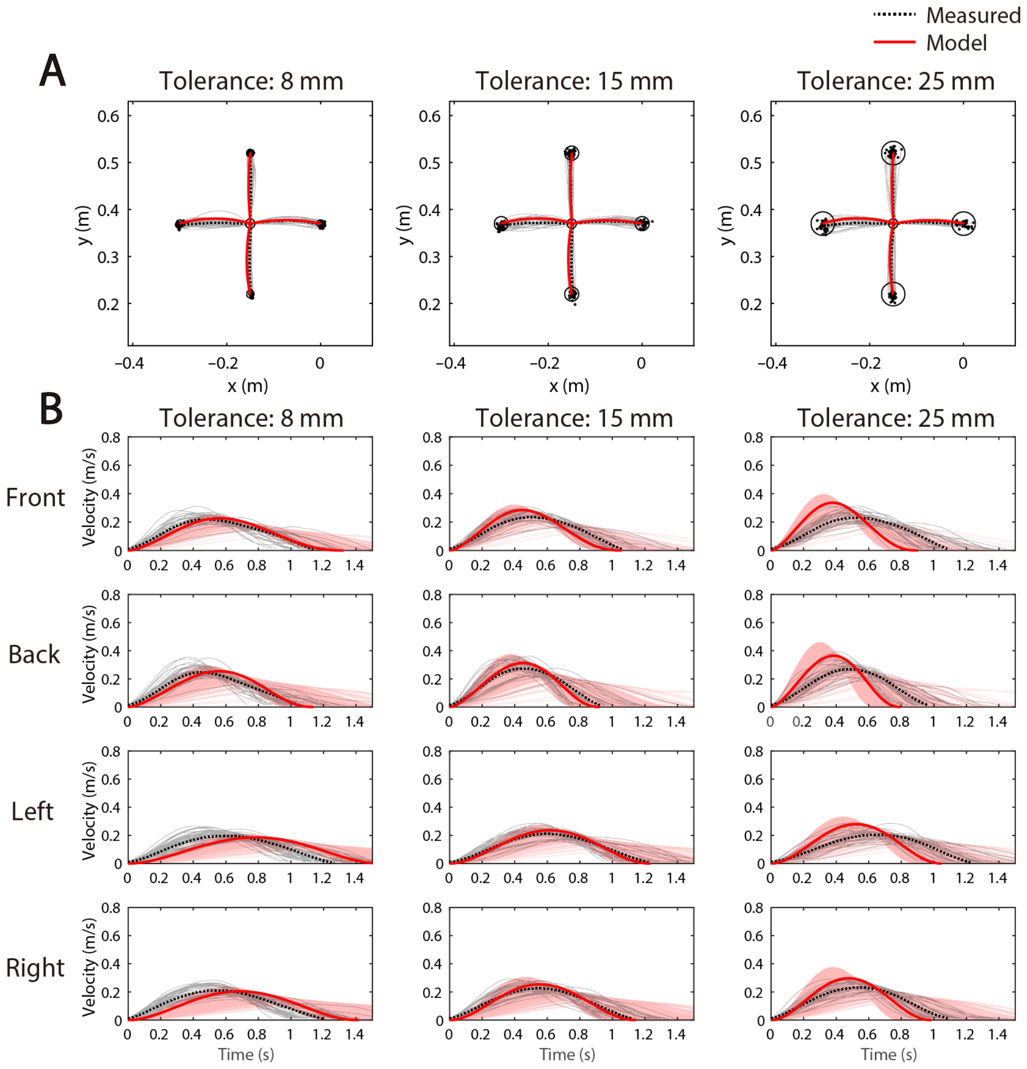

Figure S4. Subject E (Movement duration)

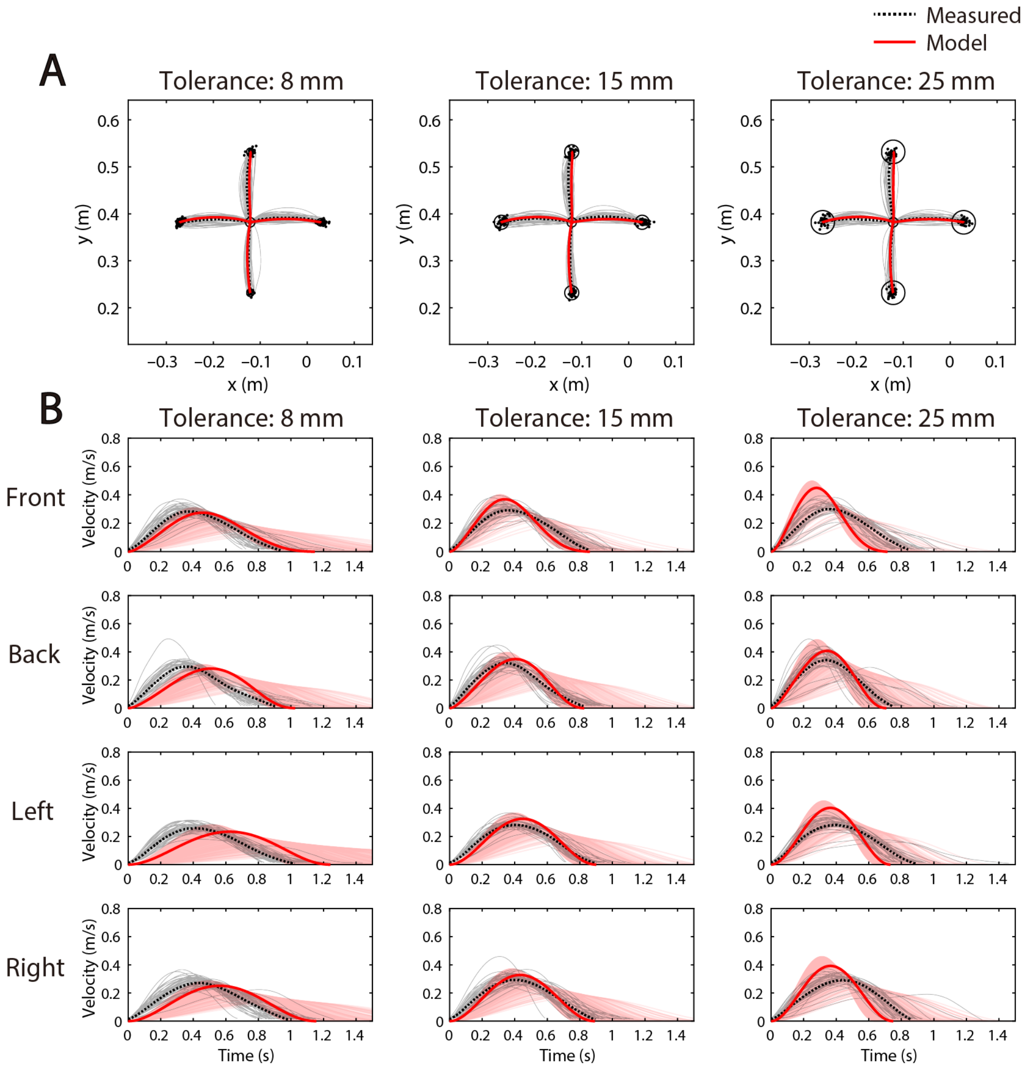

Figure S5. Subject F (Movement duration)
